# Participatory Approach to Develop Evidence-Based Clinical Ethics Guidelines for the Care of COVID-19 Patients: A Mixed Method Study From Nepal

**DOI:** 10.3389/fpubh.2022.873881

**Published:** 2022-06-27

**Authors:** Suraj Bhattarai, Anurag Adhikari, Binod Rayamajhee, Jaya Dhungana, Minu Singh, Sarun Koirala, Dhana Ratna Shakya

**Affiliations:** ^1^Department of Global Health, Global Institute for Interdisciplinary Studies (GIIS), Kathmandu, Nepal; ^2^Department of Infection and Immunology, Kathmandu Research Institute for Biological Sciences (KRIBS), Lalitpur, Nepal; ^3^School of Optometry and Vision Science, Faculty of Medicine and Health, UNSW, Sydney, NSW, Australia; ^4^Department of Anatomy, B.P. Koirala Institute of Health Sciences (BPKIHS), Dharan, Nepal; ^5^Nepali Unit of International Chair in Bioethics, Dharan, Nepal; ^6^Department of Psychiatry, B.P. Koirala Institute of Health Sciences (BPKIHS), Dharan, Nepal

**Keywords:** COVID-19 ethics, health emergency, clinical ethics, pandemic, preparedness, health for all

## Abstract

During health emergencies such as the COVID-19 pandemic, healthcare workers face numerous ethical challenges while catering to the needs of patients in healthcare settings. Although the data recapitulating high-income countries ethics frameworks are available, the challenges faced by clinicians in resource-limited settings of low- and middle-income countries are not discussed widely due to a lack of baseline data or evidence. The Nepali healthcare system, which is chronically understaffed and underequipped, was severely affected by the COVID-19 pandemic in its capacity to manage health services and resources for needy patients, leading to ethical dilemmas and challenges during clinical practice. This study aimed to develop a standard guideline that would address syndemic ethical dilemmas during clinical care of COVID-19 patients who are unable to afford standard-of-care. A mixed method study was conducted between February and June of 2021 in 12 government designated COVID-19 treatment hospitals in central Nepal. The draft guideline was discussed among the key stakeholders in the pandemic response in Nepal. The major ethical dilemmas confronted by the study participants (50 healthcare professionals providing patient care at COVID-19 treatment hospitals) could be grouped into five major pillars of ethical clinical practice: rational allocation of medical resources, updated treatment protocols that guide clinical decisions, standard-of-care regardless of patient's economic status, effective communication among stakeholders for prompt patient care, and external factors such as political and bureaucratic interference affecting ethical practice. This living clinical ethics guideline, which has been developed based on the local evidence and case stories of frontline responders, is expected to inform the policymakers as well as the decision-makers positioned at the concerned government units. These ethics guidelines could be endorsed with revisions by the concerned regulatory authorities for the use during consequent waves of COVID-19 and other epidemics that may occur in the future. Other countries affected by the pandemic could conduct similar studies to explore ethical practices in the local clinical and public health context.

## Introduction

The successive waves of coronavirus disease (COVID-19) pandemic hit the national health systems of countries worldwide, directly disrupting their capacity and resources (1). The exponential COVID-19 cases, during the 2020 and 2021 waves, overwhelmed the health facilities in Nepal too (2, 3). With a meager number of 512 ventilators and 1,180 intensive care unit (ICU) beds across the country, the availability of bedside care for critical patients was severely compromised (4). As of 30 December 2021, there were nearly seven thousand active cases of COVID-19 in the country and over 11 thousand people had died, and during the second wave, due to the increased influx of patients with Omicron variant, both public and private hospitals had to operate at their full capacity (5–8).

During the first and second waves of pandemic, the surge of patients in healthcare facilities of Nepal resulted in the breakpoints after which, the patients had to be asked to return home without treatment (9, 10). The Nepali government was not able to scale up free SARS-CoV-2 testing services across the country, which resulted in the shift in testing through private laboratories where tests were rather expensive for a low-income families (11). The pandemic also created logistic and management challenges for health workers (12).

A high influx of the poor patients caused severe delays in testing as well as hospitalization of confirmed cases, resulting in high mortality rates (13). Some of the patients who arrived at the hospital were financially crippled, but at the same time, hospital admission charges went up to 50–200 USD per day and 100–300 USD per day in public and private hospitals, respectively (14). As a consequence, some healthcare workers had to compromise the quality of healthcare to their patients. The existing national health insurance program, which does not cover the majority of the needy population nor provides satisfactory healthcare to the enrolled, did not support COVID-19 care either (15).

COVID-19 created resource scarcity not only in Nepal but globally, which disrupted the existing patient management protocols and brought public healthcare ethics challenges (5, 16, 17). When resources are not sufficient during pandemics, the protection of a larger population gets more priority compared to individual treatment and care (18). In Nepal, an unequal geographical distribution of healthcare facilities and a longstanding shortage of trained manpower affected health service delivery (19).

Ethical challenges complicated the pandemic response in many countries (16). In the Nepali model of COVID-19 response, the ethical challenge faced by front-line workers is unknown and has not been studied yet (20). There is no baseline information about the nature and dynamics of ethical issues that are directly stemming from a patient's financial roots, and more importantly, we do not know how healthcare workers are addressing these ethical challenges at the ground level in the background of weaker health systems. We hypothesized that the ethical decisions for clinical management of COVID-19 patients in the designated hospitals are based on the existing government issued guidelines such as interim clinical guidelines for the care of COVID-19 patients; infection prevention and control guidelines, and professional ethical guidelines during the COVID-19 pandemic, which are inadequate for addressing all ethical challenges (15, 21). Moreover, these guidelines were prepared by a group of experts, without taking input or feedback from the clinical end-users, nor addressing ethical dilemmas they would face while providing care to the poor and vulnerable. In contrast, the present study used the bottom-up approach—information collected from the end-users followed by inputs from the experts, with further opportunities provided to the end-users to contribute and feedback on the guideline drafts.

At various national forums and through the media, many frontline clinicians highlighted an urgent need for clinical ethics guidelines focused on health emergencies. Therefore, an idea of “participatory research” was developed by the study team, who, then, contested for the global award announced by the World Health Organization, Health Ethics & Governance Unit through the Public Health Emergency Preparedness and Response Ethics Network (PHEPREN) in 2020. It was expected that the research findings and the end product, i.e., ethics guidelines, would be endorsed with revisions by the concerned medical regulatory authorities in Nepal.

This study, in particular, aimed to develop a guideline to address syndemic ethical dilemmas during the clinical care of SARS-CoV-2 infected population who are unable to afford standard care and to explore the opinions and views of frontline health workers, health experts, and relevant stakeholders regarding ethical dilemmas during clinical care of financially troubled COVID-19 patients in the country.

## Methodology

### Study Design

This is a mixed method study conducted in the government designated COVID-19 treatment hospitals in the Kathmandu valley and among the key stakeholders in pandemic response in Nepal. The study was conducted between February and June 2021, in collaboration with the Nepal National Unit of the UNESCO Chair in Bioethics, which is located in B.P. Koirala Institute of Health Sciences (BPKIHS), Dharan, Nepal.

### Study Participants

Fifty healthcare professionals consisting of specialist doctors, medical officers, nurses, and health assistants from 12 hospitals designated for COVID-19 treatment (six public, six private) were enrolled for quantitative and qualitative data collection. This sample size reflects the minimum of four healthcare professionals from each hospital enrolled in a time constrained situation in a pandemic, which is six-fold of what is considered the minimum in a Delphi method. Additionally, 15 expert individuals were interviewed to collect additional qualitative data. The stakeholders engaged in this study were divided into five major groups: frontline COVID-19 responders (*group A*) and representatives of the government of Nepal (*group B*), humanitarian bodies (*group C*), regulatory bodies and professional associations (*group D*), and health specialists (*group E*). Details of each stakeholder group are given in the table below (Table 1).

**Table 1:** List of stakeholders who participated in the study.

**Group**	**Stakeholders group**	**Participated stakeholders**
A	Medical workforce	Frontline COVID-19 responders from 12 selected hospitals
B	Government of Nepal	Ministry of Health and Population - Health Emergency Operation Center (HEOC)/ Health Emergency Disaster Management Unit (HEDMU), COVID-19 Crisis Management Center (CCMC)
C	Humanitarian bodies	Nepal National Unit of UNESCO Chair in Bioethics (BPKIHS)
D	Regulatory bodies and professional associations	Nepal Medical Council (NMC), Nepal Nursing Council, Nepal Medical Association, Nepal Nursing Association, Nepal Critical Care Society, Nepal Geriatric Society
E	Other health specialists	Emergency and Family medicine, Anesthesia, Child health, Women's health, Mental health, Public health, Infectious diseases, Medical education, Medical ethics

### Study Tool

A study questionnaire was developed by the study team (Supplementary File 1) to collect participants' socio-demographic data and measure the ethical challenges faced by them (using a Likert scale) during the first wave of COVID-19 (March 2020 to January 2021). Ethical dilemmas/challenges were categorized into four levels: (a) contextual challenges (resource scarcity and patients' socioeconomic status), (b) challenges in the decision making process, (c) provider-related challenges, and (d) patient-related challenges (22). The quantitative study was followed by the qualitative components: interviews with the key informants, followed by discussion among stakeholder groups (Delphi process) to prepare a list of ethical dilemma situations and potential solutions (23).

### Research Activities

#### Activity I: Identification of Stakeholders and Initial Interaction to Introduce the Problem and Research Questions

**Figure 1:**
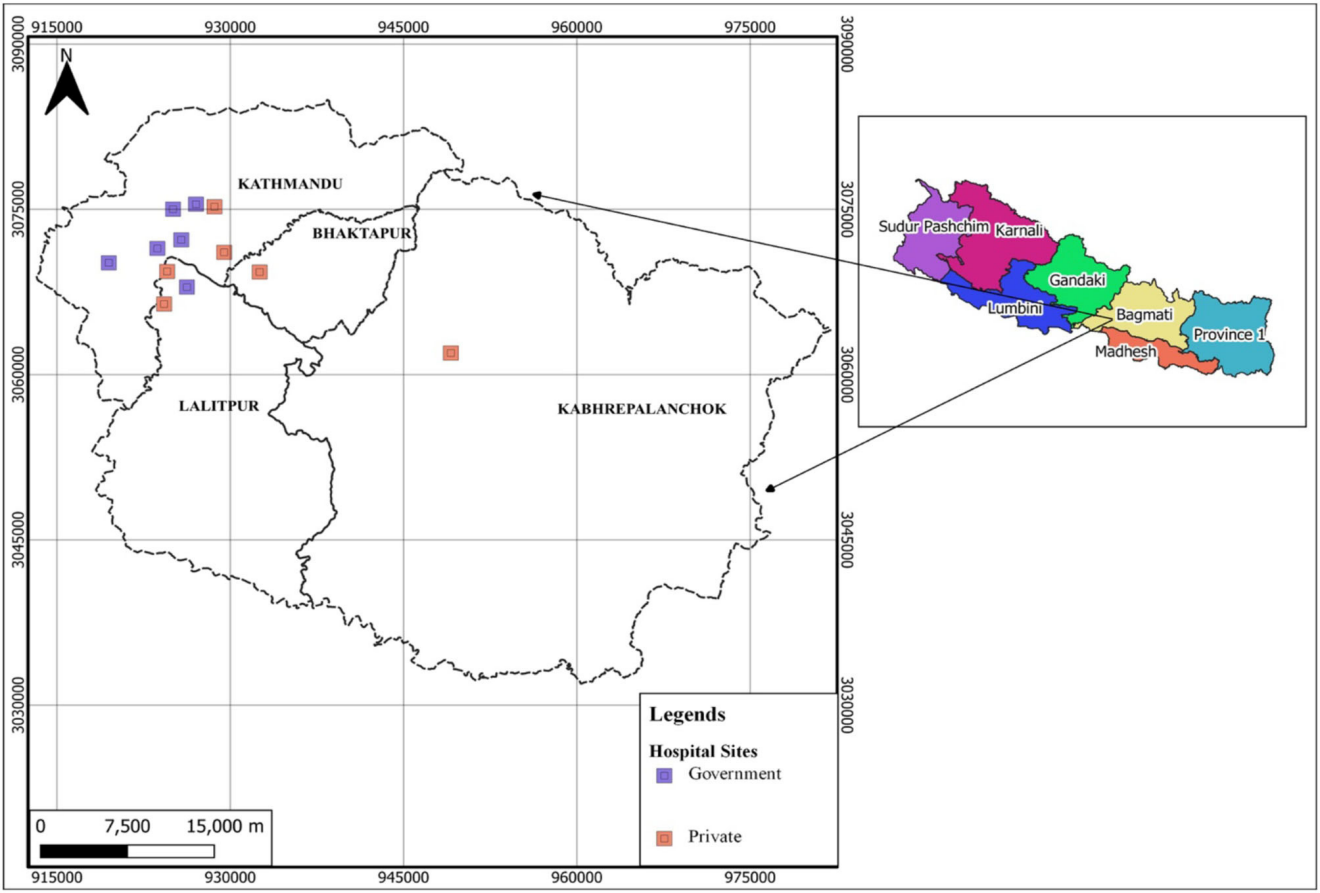
Geographical information system (GIS) map showing the location of 50 health professionals recruited from 12 COVID-19 hospitals, created using ArcGIS (Esri GIS, California, USA).

We identified 50 healthcare workers who were working in COVID-19 hospitals and were also members of professional organizations mentioned in Group A (Table 1). We approached 12 tertiary hospitals located in Bagmati province (Figure 1), that were treating COVID-19 patients, given the sustained surge of cases in these facilities. Study participants were physicians (emergency, critical care, and medical officers), nurses, paramedics, and public health officers, where gender distribution was accounted for. They were selected through the recommendation of COVID-19 focal persons in each hospital. A formal invitation to the session was sent to them along with a participant information sheet and consent form. For those agreeing to participate, a set of semi-structured questionnaires for discussion was sent by email a day before the scheduled session.

Twelve facility-based groups were formed out of 50 nominated individuals. A 30-min virtual interactive session (recorded version) was conducted for each group, and the session was facilitated by one of the co-investigators. The interactive session with one group was blinded to the other groups. Five-open ended questions (which represented the major ethics-related themes: equity, justice, transparency, patient's autonomy, and professional hierarchy) were discussed in detail. All recorded responses were anonymized before data analysis, which is described in the data management section.

#### Activity II: Key Informant Interviews With Stakeholder Groups

Interviews with the experts were scheduled to discuss the ethical challenges documented from an activity I (see above) and the potential solutions to context-specific challenges. Fifteen experts were identified (five from group A, two from group B and group C each, and six from groups D and E). A formal letter was sent to the president or the director of each institute listed in groups B–E, with a request to nominate these experts. Of 15 interviews, 11 were conducted in-person whereas 4 were conducted virtually. Two or more investigators facilitated each interview. The information generated in the form of an audio draft of around 30-min interview was transcribed by the project team, then sub-categorized into five dilemma situations (described in the results section as pillars) along with their solutions, altogether developing a draft of clinical ethics guideline.

#### Activity III: Expert Review of COVID-19 Clinical Ethics Guidelines

This session recalled 10 experts from previous sessions (Activity I–II) and recruited five new participants from groups A–D (Table 1) following similar selection methods as described above. The additional informed consent form was added to cover this session. The draft guideline was emailed to all participants 48 h prior to the review meeting. The session was conducted in-person, and moderated by an investigator. All feedback were audio-recorded and all suggestions were incorporated into the draft guidelines.

#### Activity IV: Dissemination of COVID-19 Clinical Ethics Guidelines to End Users for Feedback and Orientation

The near final version of clinical ethics guidelines along with a standard feedback form was sent out by email to 50 end-users from an activity I. All of them responded. The same feedback form was also used to measure the impact of ethics guidelines (based on scores on the Likert scale) reflecting upon the practicality and usefulness of the guidelines, as well as the barriers to its uptake and application in pragmatic settings. Additionally, a half-day virtual orientation session was organized to orient other 20 end-users of the guidelines, who were identified through the recommendation of COVID-19 focal persons of the designated hospitals. The session was facilitated by two investigators. During the session, we used the feedback questionnaire form (Supplementary File 1) to collect participants' feedback on COVID-19 clinical ethics guidelines as well as the feedback on the effectiveness of the orientation program.

### Data Management and Translation

All the virtual recordings were done via *Microsoft teams (version 4.8.19.0)* and face-to-face meetings were recorded utilizing *Philips DVT-4110*. Each recording was transferred to the project computer as an audio file. After deidentification of the audio files, they were transcribed, and the original file containing audio recordings was stored in the project computer as an encrypted password protected item. As all of the interactive sessions and interviews were conducted in the Nepali language, all of the data were translated into English version during analysis.

### Ethical Approval

Before data collection, ethical approval was obtained from the Institutional Review Committee of the B.P. Koirala Institute of Health Sciences (*Ref. No. 497/077/078-IRC, Code No. IRC/2099/021*) and the WHO COVID-19 Research Ethics Review Committee (*Ref. No. CERC.0088, 3/3/2021*). A written informed consent was provided by the study participants for their participation in the respective activity.

## Results

Fifty frontline healthcare professionals were recruited in this participatory study from 12 different COVID-19 treating hospitals (Figure 1). The median age of the participants was 32.5 years [SD ± 6.14, (IQR: 28 to 34.75 years)] and 50% were female. Of all participants, 40% were specialist doctors (internal medicine, infectious disease, anesthesia, and critical care), 20% were nurses, 13.3% were junior doctors, and 10% were health assistants. Half of the participants (53.3%) were from private COVID-19 hospitals and 36.7% had >10 years of work experience in their related fields. The participants' score (Likert scale) for ethical challenges confronted during the COVID-19 pandemic (March 2020 to January 2021) was not significantly (Mann–Whitney *t*-test) associated with participants' gender and primary work institution (private vs. public).

Out of 15 experts interviewed on a one-to-one basis, three were female. All of them held the leadership position at their respective institution, as mentioned in Table 1.

**Figure 2:**
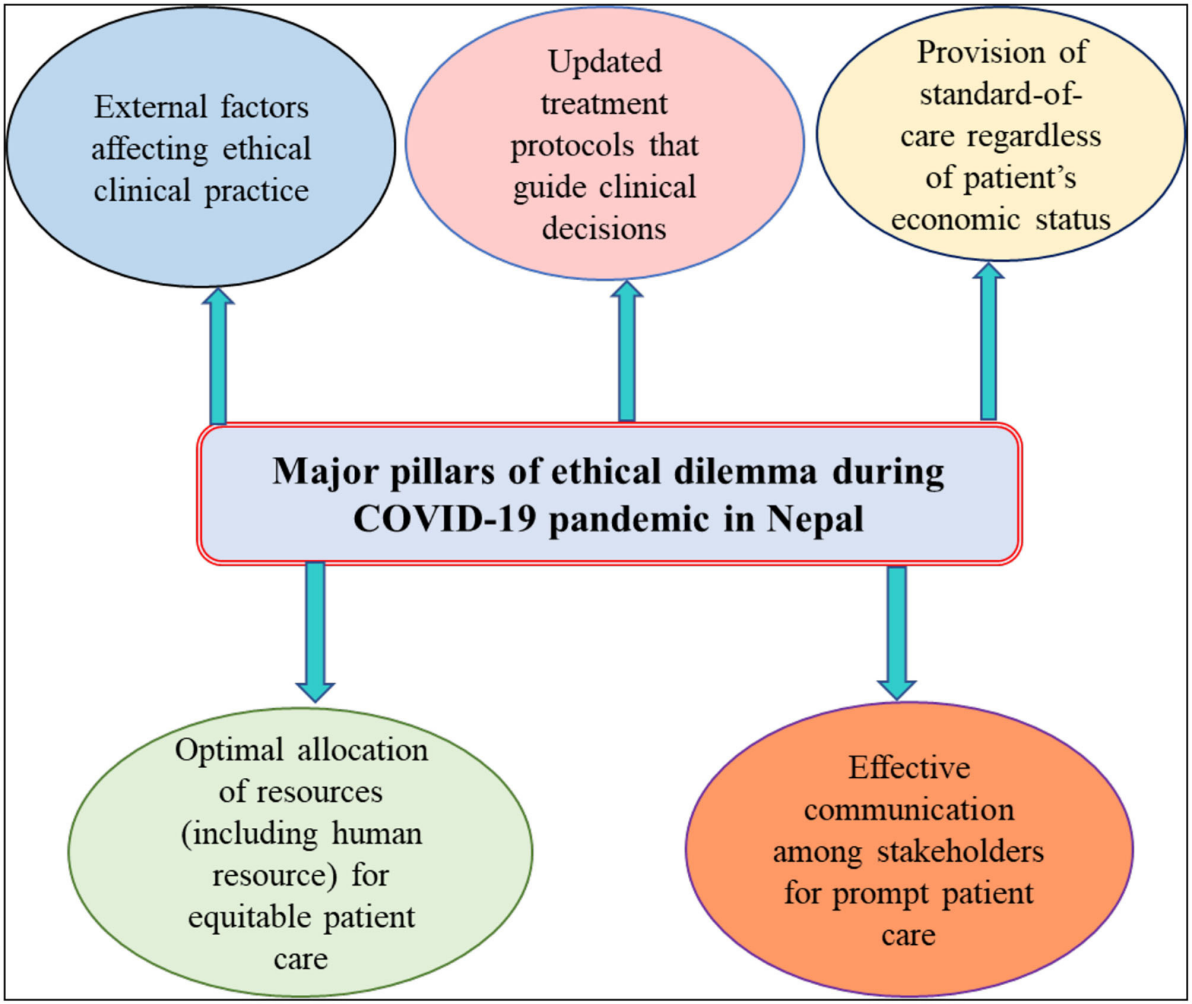
Major ethical dilemmas experienced or observed by healthcare professionals of Nepal during the COVID-19 pandemic.

The major findings of this participatory study are summarized below under five sub-sections considered as the five pillars of clinical ethical practice during public health emergencies (Figure 2).

### Pillar I: Optimal Allocation of Resources for Equitable Patient Care

It was observed that lack of medical resources including qualified human resources is a key problem during health emergencies such as COVID-19. Existing gaps in the medical curriculum about public health emergencies and their management is another issue in Nepal. Other recurrent issues faced by the healthcare facilities during the COVID-19 pandemic included: proper allocation of trained clinical staff for COVID-19 care, procurement of consumables including personal protective equipment, sanitizers, etc., and choosing suitable diagnostic methods for case detection. Other commonly reported issues included: dilemmas in decision making regarding resource allocation for patients' vs. healthcare workers, the challenge in deploying a non-COVID-19 workforce for COVID-19 care, and looming shortage of health and care workers due to quarantine and isolation requirements post-exposure. The introduction of evidence-based courses on public health emergencies, emerging infectious diseases, and epidemics targeted at frontline workers was one of the recommended solutions to these issues.

Study participants advised that identification of the breakage point of resources is crucial during a pandemic as it could help hospital managers to anticipate the scarcity and means to tackle such problems during a health emergency. In addition, a defined breakage points could help to start procurement and hiring of medical resources and staff ahead of a pandemic emergencies. Especially, the local private–public partnership could be practiced for the procurement of raw materials to manage limited resources. In summary, study participants advised the establishment of new structural units to manage relevant expertise and resources promptly during health emergencies.

### Pillar II: Updated Treatment Protocols That Guide Clinical Decisions

Study participants reported different issues related to the COVID-19 treatment approach and decision making which include varying screening and testing strategies between hospitals, patient's clinical care needs vs. hospital's profit motive. On top of all, variations in institutional policies and regulations were also observed in the hospitals. These facilities were devoid of contextualized guidelines to provide efficient health services to the COVID-19 patients. As a solution, participants suggested health facilities formulate their own ethics guidelines from administrative and management perspectives for prompt and efficient response to public health emergencies. Participants realized the importance of clear and timely updated guidelines to control and regulate unapproved treatment methods such as under trial therapy and herbal medicines.

### Pillar III: Provision of Standard-of-Care Regardless of Patient's Economic Status

High fees for hospitalization, especially intensive care, in both public and private facilities were found to be the most common issue observed by all study participants. The COVID-19 patients had to suffer due to unclear guidelines and notices from the government, with frequent changes, especially about subsidized care and designated facilities for the same. Unfortunately, some patients were forced to turn away from the hospital entrance just because of their inability to pay the deposit amounts in advance, particularly in private hospitals. Most notably, healthcare professionals were deficient in the rationale behind the use of blanket therapy for the treatment of mild to severe COVID-19 patients. Study participants highlighted the importance of strictly following medical ethics and professional codes of conduct to reduce treatment disparities based on the patient's financial status.

### Pillar IV: Effective Communication Among Stakeholders for Prompt Patient Care

During the first wave of the COVID-19 pandemic, all hospitals in Nepal were required to report PCR tests conducted in their lab to the government before relaying the results to the patients. Because of this rule, many patients complained about the delay in getting PCR results and deferred treatment. The lack of clear diagnostic and treatment protocols and guidelines embellished the situation, coupled with the longstanding practice of professional hierarchy for clinical decision making.

Misinformation and infodemic about SARS-CoV-2 infection and new emerging SARS-CoV-2 variants also intensified public panic, especially among the minority indigenous groups and people with poor health literacy. Study participants agreed that up-to-date information to the public with evolving evidence disseminated through authorized channels is very important to avoid unnecessary havoc during health emergencies. On the other hand, each health professional should be made aware and well trained in scientific communication, effective use of PPE, and appropriate patient referral and follow-up mechanisms. It is also a fundamental responsibility of the hospital management to arrange relevant e-learning courses and hands-on training for all staff on a regular basis – before, during, and after health emergencies.

### Pillar V: External Factors Affecting Ethical Clinical Practice

Study participants reported undue pressure and influence from senior members of the hospital, high government officials and bureaucrats, and political figures to prioritize their family, relatives, or friends for COVID-19 care, sometimes trespassing the in-patient units and ignoring the institution's infection prevention and control (IPC) measures. Similarly, some study participants experienced hurdles to get the ethical approval from regulatory bodies to conduct research related to COVID-19. The government and institution's reluctance for the recognition of COVID-19 as an occupational disease, especially for healthcare workers (HCWs), was a unique challenge noted by the study team.

A detailed explanation of ethical challenges and dilemmas experienced or observed by the healthcare professionals of Nepal during COVID-19 patient care has been given in Table 2, where a summary of possible solutions and recommendations is also mentioned.

**Table 2:** Major ethical dilemmas/challenges experienced by healthcare professionals in decision-making process during COVID-19 pandemic.

**Pillars of ethical clinical practice**	**Identified challenges**	**Possible solutions and recommendations**
**I) Optimal allocation of resources (including human resource) for equitable patient care**	1. Qualification of medical workforce and gaps in curriculum	• Introduction of evidence-based courses on public health emergencies, increase course hours/credits for emerging infectious diseases and epidemics • Mandatory aptitude test (with ethics related assessments) as a screening tool for pre-medical students • Training on IPC guidelines should be made mandatory for all healthcare workers with annual re-certification.
	2. Resources, including PPE, ventilators, ICU beds, etc. have a finite amount given they are continuously manufactured, and they must be restocked upon consumption. However, restocking during health emergency is a challenge. How can we prepare ourselves?	Breakage point of resources is different for different resources, thus, should be defined on a case-by-case basis. This definition will help to anticipate the scarcity and means to tackle it.
	3. Issues about deployment of trained clinical staffs for COVID-19 care, procurement of consumables such as PPE, sanitizers, etc. and choosing suitable diagnostic methods for case detection. How can we minimize the resource strain during health emergencies? How can we ease procurement of construction materials for establishing new COVID-19 wards or repurposing the existing wards for COVID-19 care?	• Government stakeholders as well as hospital managers should have a breakage point defined for each resource, then, they should start the procurement and hiring ahead of such point. • It would be ideal if each hospital would anticipate new structural development in pandemic so that relevant expertise and resources could be managed promptly. In Nepali setting, some of the innovative approaches were utilized for minimizing the impact of limited resources (e.g., in house manufacturing of PPE, hospital beds, oxygen plant; local public private partnership for raw material production or procurement).
	4. Given the nature of pandemic due to emerging disease, the information and guidelines may not always appear promptly especially during initial days of pandemic. Who should decide for the resource allocation in healthcare facilities?	• The best party for deciding resource allocation during health emergency is the hospital itself. • A core team including physicians, nurses, administrative personnel, and technicians with variety of experience might provide a holistic idea on resource allocation. • It is also important not to heavily engage clinicians in the decision-making process for resource allocation. However, the core team should listen to the insights and experience of frontline responders regarding resource allocation for healthcare workers and patients. • The core team should protect healthcare workers from accusation of bias in resource allocation, for e.g., favoritism for certain patients and negligence for others.
	5. How should resource allocation among patients vs.. healthcare workers be decided?	• The resource allocation decisions should not be influenced by the patient's gender, religious or political views, ethnicity, financial status etc. However, we should be aware of fact that complete elimination of all this bias is impossible, so we should work more on minimizing the disparities and discrimination. • Decision on need-based resource allocation could be implemented. For example, PPE allocation will have different rationale compared to ICU beds and ventilators. Hence, creating a subtopic on “resources” and defining the breakage point for all resources is necessary. • Resource allocation process should also cater to the needs of healthcare professionals regardless of their hierarchy in the institution.
	6. Challenge in deploying non-COVID-19 work force for COVID-19 care	A role model based leadership is optimal and can impact positively for motivating the existing workforce for their smooth transition to COVID-19 care.
	7. Shortage of health-care professionals for COVID-19 care due to quarantine and isolation requirements after exposure to infected patients	• Deployment of highly trained but inactive health care workforce into the frontline would be an alternative of managing human resource at the time of health emergencies. • Inclusion of newly trained medical students and interns in the front responders' list could help fill the human resource gap. • Medical or nursing licensing procedure can be eased to pool trained manpower for quick deployment during health emergencies.
II) Updated treatment protocols that guide clinical decisions	1. Screening, diagnostic and testing tools/strategies vary between hospitals	Steps for standardization of tools/strategies: collection and review of global practices, guidelines, and strategies – select those most suitable for the local context – make uniform tools that could be applied in all types of hospitals – adopt the tools/strategies as pilot followed by nationwide roll out.
	2. What is the optimal timeline and duration of in-hospital treatment of COVID-19 patients?	Need of locally contextualized guidelines and protocols regarding when to end quarantine/ isolation/ ICU/ hospital care for infected patients.
	3. Pandemic response related institutional policies and regulations vary between hospitals	Need of uniform policies and regulations in all private hospitals across the nation regarding pandemic response.
	4. Patient's clinical care needs vs. hospital's profit motives (especially in private hospitals)	Need of hospital ethics guidelines (from admin/ management perspectives).
	5. Home nursing care provision for COVID-19 patients	• Home care should be permitted for registered institutions only, that too for preventive and promotive care only. • Discourage this service unless there is a code of ethics; there should be regulations for nurses who want to provide home nursing services with their individual discretion or through authorized channels.
	6. Use of Robot nurses for COVID-19 patients	Need of protocol and regulations for/against the use of Robot nurse, although it has been conditionally approved by the MOHP as a trial service.
	7. Use of under trial drugs and procedures (such as remdesivir, dexamethasone, ivermectin, CPT)	Need of clear and timely guidelines to regulate use of unapproved treatments (such as CPT with measure of neutralizing antibodies).
**III) Provision of standard-of-care regardless of patient's economic status** *Applicable to both public and private service providers*	1. Unclear guidelines and notices, with frequent changes, about treatment subsidies for COVID-19 infected individuals and designated centers for the same	Autonomy should be given to the hospital management in deciding treatment for the poor. Government authorities, in turn, could revitalize existing universal medical ethics and professional codes during crisis and support formation of a social welfare committee in each hospital to address poor patient related issues.
	2. Drugs under trial (such as remdesivir) were not available in all hospitals and to all patients	• All hospitals meeting the standards for clinical research should be enrolled into clinical trials and their names should be circulated to all treatment facilities. • Physicians working elsewhere could coordinate transfer of patients to the designated research hospitals so that they can be enrolled into trial.
	3. Some drugs and procedures (remdesivir, steroid, plasma therapy) were not accessible to poor patients due to unregulated price hike and artificial shortage	• Treating physicians, hospital management or staff welfare committee (SWC) could coordinate/lobby with national research and regulatory bodies and pharmaceuticals to ensure poor patient's access to emergency medicines at affordable price. • Hospitals could write a formal letter to the philanthropists and donor organizations requesting in kind contribution to the poor patient fund.
*Public service providers*	4. Cumbersome paperwork for patients to qualify or self-declare poor status to take subsidies and benefits	Treating physicians should continue providing care to the likely poor patients until their paperwork is complete. Physicians can later confirm the poor status of patients through hospital management or SWC, whenever the required documentation is complete.
*Private service providers*	5. High cost of in-patient care, especially intensive care (ICU)	• Hospitals should admit only those patients who require hospitalized care but ensure continuation of telehealth services to mild cases, transfer asymptomatic or mild cases to the government-designated isolation centers. • Government could begin market survey to make treatment packages (including individual drug prices) uniform and reasonable across all health facilities (public or private).
	6. Some poor patients were turned away from the hospital gate just because of inability to pay deposit amount in advance	• Treating physicians should strictly follow medical ethics and professional codes of conduct. • Clauses of hospital ethics should be regulated by SWC. • Hospital management should not encourage unethical practices and disparities based on the financial status of patients.
	7. Clinicians as the owner of hospitals or taking the leadership role in the management could have influenced pandemic response and clinical decision-making process	Remove selection bias while nominating SWC members.
	8. Dilemma among health care professionals around patient needs vs. patient or relative's request vs. professional ethics. How can it be minimized?	Healthcare professionals should review the rationale and evidence behind use of sophisticated and non-recommended tests such as HRCT Lung (patient need vs. patient/family request vs. professional ethics), use of blanket therapy for treatment of mild to severe patients (which compels patients to pay out of their pocket) such as steroids, broad spectrum antibiotics, antifungal, and other repurposed drugs and therapy).
**IV) Effective communication among stakeholders for prompt patient care**	1. Each hospital (public or private) with RT-PCR lab facility was required to report to the government before relaying test results to the patients. Many patients complained about the delay and their ignorance about the next steps	The government should respect the autonomy of service provider and patient with regards to test reports. Along with the test result, it would be better to disseminate IPC information and the next steps for the individuals who deposited their specimens for COVID-19 testing (regardless of test result).
	2. Unclear treatment guidelines and protocol	• Treating physicians may continue patient care based on the evidence and experience while remaining vigilant to the new directions from the government. • The government should allow clinical autonomy to physicians until a centralized evidence synthesis institution is established.
	3. Several questions asked by patients/families could not be answered by the clinicians due to lack of evidence.	It is the responsibility of a qualified clinician to remain up to date regarding evolving evidence and share any new information to the patients in a lay language. Treating physicians should provide updates to the patients/ families on a regular basis. Ensure adequate care contact time between service providers and patients/families.
	4. Professional hierarchy affected clinical decision-making process	• Experienced and qualified junior professionals should be given equal autonomy even under no or minimal supervision of senior professionals to save time while providing clinical care to the needy patients during health emergencies. • Medical or nursing councils should remain standby to resolve any pertinent issues regarding hierarchy that might affect optimum clinical care.
	5. Misinformation and infodemic circulating in free social media platforms; Social stigma about COVID-19; Poor access to the right and adequate information, especially for people with digital illiteracy and those from minority ethnic groups	• Public media platforms should be given to the genuine experts and non-experts should be restricted from sharing unsolicited opinions. • Rapid communication groups or social media pages may be formed to run instant debates and discussion on emerging topics. • All stakeholders should disseminate positive message through social channels such as radio, daily newspapers, TV, etc. to reduce misinformation and stigma. • Infodemic about unapproved tests, treatment, and prevention strategies (for example, Ct value information was not need in RT-PCR report) should be discouraged by the government and professional societies. • Government's communication strategy should prioritize translating and disseminating all relevant public information in all local languages to reach ground level communities.
Communication amongst service providers	6. Because COVID-19 was an emerging disease, there was a dearth of information and updates even from authentic sources.	• It is the responsibility of a qualified clinician to remain up to date regarding evolving evidence. • Hospital should identify a dedicated staff who can track all relevant sources of information, collate up-to-the minute updates regarding emerging disease that are available on the internet, and disseminate the findings to the clinical and management team on a daily basis.
	7. Inadequate information regarding service availability in COVID-19 treating hospitals (especially oxygen beds, ICU service, ventilators) which hampered timely and safe referral/transfer of moderate to severe patients	• Mapping of available services through government or non-governmental authorities (such as HEOC, NMA) with hourly updates, public dissemination of updated contact list of service providers in each hospital, and instant communication through social media platforms such as Viber group/WhatsApp group/Facebook group.
• Dedicated and qualified healthcare as well as managerial personnel could be recruited for Hotline services offered by the government.	8. Lack of proper information regarding effective use of PPE (especially doffing) while providing care to infected patients and the follow-on steps (whether or not required to stay on isolation after seeing infected patient, timeline for return to care, degree of precautions to be taken at home). Limited training slots for healthcare providers, so not all staff could receive the training.	• There should be a provision for continuous and on-demand training opportunities for all levels of healthcare providers. • Training should be provided in a simple and understandable language with hands-on learning. • Hospital management and senior professionals should provide clear guidance and directives to the junior staffs.
**V) External factors affecting ethical clinical practice**	a. Administrative hassle for research ethics approval and unclear rational behind selection/designation of research centers	Expedited and free of cost processing of research proposals submitted to the ethical review committees. Designate research centers based on qualified human resource, quality of patient care with ICU back up, and availability of advanced technology.
	b. Gender related incidents and violence in isolation centers	The government should manage supervision of isolation centers from violence, gender, and GBV perspectives.
	c. Undue pressure and influence from higher officials and political figures for priority care of their families, relatives, and friends	• Senior members of the hospital, government's high-ranking officials and politicians along with their cadres should follow IPC measures when they visit hospital for whatsoever reason. • They should not influence the priority setting of COVID-19 care to the infected patients. • Concept of “health equity” and “health for all” should be understood by everyone.
	d. Healthcare providers were prone to contracting infection due to exposure at workplace	Recognition of COVID-19 as occupational disease, especially for HCWs.

*GBV, gender-based violence; PPE, personal protective equipment; ICU, intensive care unit; SWC, Staff Welfare Committee; CPT, convalescent plasma therapy; IPC, infection prevention and control; Ct, threshold cycle; HCWs, healthcare worker*.

## Discussion

Health emergencies such as the COVID-19 pandemic emphasize the importance of clinical ethics which values the greater good of the whole society rather than individual demands and rights (24–27). The COVID-19 pandemic has raised various ethical concerns, especially in the low to middle-income countries (LMICs). Most of the ethical concerns are around sharing and allocation of medical resources, triaging and care of the sick patients, preparedness and readiness of the health facilities and overall health systems, information sharing mechanisms, intellectual property rights, community engagement for health emergency decisions, and inequity in healthcare (16, 28). This end-user participatory study developed evidence-based ethical guidelines for the care of COVID-19 patients in Nepal, based on the major ethical dilemmas confronted by study participants which can be broadly categorized into five sections: (i) rational allocation of medical resources; (ii) appropriate treatment protocols to guide clinical decisions; (iii) patient's economic status affecting optimal treatment and care; (iv) effective communication among stakeholders for better healthcare service; and (v) undue pressure and IPC breach by political and bureaucratic figures.

As in other countries, the resource allocation process in Nepal was exacerbated by the shortage of essential medical products including PPE, ventilators, beds, oxygen, and medicines, which created a high level of insecurity and uncertainty among COVID-19 patients and caregivers (16, 29–31). During the pandemic, appropriate criteria and norms could have been established for the distribution of already scarce critical care supplies on a case-by-case basis. Each hospital could have established a rapid response team comprised of clinicians and hospital managers which could provide the right direction for resource management. A transparent and open communication amongst hospital staff is also crucial during a health crisis to make quick decisions for a scientific allocation of resources including the health workforce (32).

Most importantly, resource allocation decisions should not be influenced by ethnicity, gender, religious or political view, and the financial status of the patients (33). For ease of allocation, resources can be divided into as many parts as possible, so that need-based decisions could be implemented. For example, the rationale for PPE allocation could be different compared to intensive care ventilators. Thus, creating a subcategory of the resource and then defining the point of breakage for each subcategory would be necessary for prompt and scientific allocation (34).

Like other countries, Nepal also experienced the challenge of repurposing the non-COVID-19 healthcare workforce for COVID-19 care (12, 18, 35). A looming shortage of healthcare workers was worsened due to strict quarantine and isolation obligations after minimal exposure to the infected patients. To mitigate the shortage, this study suggests the identification and deployment of a highly trained but clinically less active healthcare workforce at the time of health emergencies. Moreover, it would important to advocate for medical education reform as ethics education or training is missing in the medical curriculum of Nepal (36, 37).

Lack of standard protocols for screening and testing of COVID-19 suspects, lack of clear treatment guidelines, and dilemma about prognosis scoring of critical patients were found to be the major ethical challenges faced by the majority of physicians. A similar scenario was prevalent in India, South Africa, the UK, and globally (16, 35, 38). Particularly, it was unclear when to end the quarantine, isolation, or hospitalization requirements, not only for the patients but also for the exposed healthcare workers. There was also a lack of clear and updated guidelines regarding the use of unapproved COVID-19 treatments such as remdesivir and convalescent plasma therapy (39). On another hand, participation of health institutions in large research was affected due to a lack of clear and contextual health emergency-focused “research ethics” guidelines (40, 41).

The financial motives of some of the large private providers also overshadowed the optimal clinical care needs during the COVID-19 crisis, as some patients were forced to struggle with high treatment costs (42). The availability of ICU beds surpassed the epidemic intensity and its simple solutions, such as the transfer of ICU patients from central to regional hospitals, were not implemented. Surprisingly, private hospitals and nursing home facilities did not receive positive feedback and support from the policy-makers despite their interest and capacity to initiate care and treatment of COVID-19 patients. The study participants suggested the utilization of nursing or care home facilities to provide appropriate and safe care for COVID-19 recovered patients who need short-term or long-term residential care (43).

Frequent changes in the government's work plan and directives regarding subsidies to the poor and vulnerable COVID-19 patients were another reason for the ethical dilemma that the clinicians faced. This study observed that the formation of a “social welfare committee” in each hospital, particularly during a health crisis, could be a fast-track to addressing the issues of the poor and vulnerable patients. Tireless paperwork required for the patients to qualify or self-declared “poor status”, even to get minimum benefits became a burden for the majority of patients. There was a need for a proper channel that could have coordinated among hospital management, treating physicians, regulatory authorities, and pharmaceutical bodies to ensure the poor's access to basic and emergency services as well as medicines at affordable prices. The study participants also advised the government to conduct a market research to estimate price variations across health facilities, then develop a uniform and consistent treatment and benefits packages (44).

The study also highlighted the need to reinforce the clinical workforce to strictly follow medical ethics and professional codes during patient triage and treatment. It was advised that the clinicians should admit only those patients who genuinely require in-patient care, but at the same time ensure the continuation of telehealth services for ambulatory patients wherever possible. Asymptomatic or mild patients could be transferred to the government-designated isolation centers to minimize overcrowding in tertiary level COVID-19 designated hospitals. It should be mandatory for everyone to follow the hospital's IPC measures, even the senior members of the hospital, government's high-ranking officials, or political leaders, whenever they enter the facility regardless of the purpose. In prescribing behavior, the physicians could be advised not to use blanket therapy approach for treating mild to severe COVID-19 patients, as a method not only to reduce the patient's out-of-pocket expenses but also to minimize the risk of drug resistance (45).

Shared decision-making and open communication among stakeholders can help improve patient care at the time of a health crisis (46), but both methods were lacking in the healthcare facilities of Nepal during the COVID-19 pandemic. The civil society organizations, national/international non-government organizations, and local/provincial governments could have played a role in solving ethics-related issues by utilizing their pre-established coordination and communication channels. Few examples of effective communication which could benefit the patients and their relatives at the time of emergency are hourly updates on the availability of essential health services (e.g., oxygen, isolation beds, ICU beds, ventilator) at public and private health facilities, updated contact list of on-duty service providers, and mechanism for instant communication through social media channels. On the other hand, it is important to verify the information and updates based on the evidence available and disseminate them through authorized communication channels to avoid unnecessary public havoc during health emergencies (13).

It was also observed that professional hierarchy in an institution affected the clinical decision-making process and delayed care of COVID-19 patients. As a solution, qualified junior health professionals could be allowed equal autonomy to provide clinical services to the patients, under minimal supervision of seniors, at the time of crisis (47).

This participatory study enhanced the capacity of end-users, i.e., frontline clinicians and healthcare workers, to some extent, which will help them address ethical issues that may arise during routine and emergency clinical management of the patients. And, to sustain the mechanism, continuous training should be provided to all healthcare workers, regardless of their position or level, to update them about rapidly changing clinical scenarios during a health emergency.

## Conclusion

The COVID-19 pandemic posed a wide range of challenges to the health systems of Nepal, but also an important prospect to reflect and develop new methods and models of delivering clinical services in an ethical way, which is essential at the time of public health emergencies. Our findings suggest that the majority of clinical ethics dilemmas while providing health services to the needy patients were stemming from resource allocation, treatment protocols for clinicians, patients' socio-economic status, communication strategy, and political/bureaucratic support. We suggest that a co-design bottom-up approach and synergistic model of care might be helpful for rationing limited resources and priority setting to ensure quality clinical care for all patients. There might be a need for an overhaul of the health infrastructure on par with the preparation drill for pandemic-like situations in each health institution to minimize the potential ethical dilemma. In addition, this living clinical ethics guideline, which has been developed based on the local evidence and case stories of frontline responders, is expected to inform the policy-makers as well as the decision-makers positioned at the concerned government units. The guidelines could be endorsed with revisions by the concerned regulatory authorities for the use during consequent waves of COVID-19 and other epidemics that may occur in the future. The Nepal National Unit of UNESCO Chair in Bioethics, a study collaborator, could facilitate the implementation and routine update of the guidelines by key health system actors, such as the social security division at the Department of Health Services and the Health emergencies unit at the WHO country office. Learning from the findings of this study, other countries affected by the pandemic could conduct similar studies to explore ethical practices in the local clinical and public health context.

## Limitations

Standard clinical ethics guidelines are important, but these are not the only solutions to ensure quality health services for the poor and vulnerable populations. Overall health systems of the country need to be strengthened to provide health coverage to all people regardless of their financial status. An ethical practice of health service delivery should be a joint venture of health service providers in both public and private sectors, national health insurance and social protection programmes, and relevant regulatory bodies of the government.

## Data Availability Statement

The original contributions presented in the study are included in the article/Supplementary Material, further inquiries can be directed to the corresponding author/s.

## Ethics Statement

Ethical approval for the study was obtained from the Institutional Review Committee of BPKIHS (Ref. No. 497/077/078-IRC, Code No. IRC/2099/021) and the WHO COVID-19 Research Ethics Review Committee (Ref. No. CERC.0088, 3/3/2021). The participants provided their written informed consent to participate in this study.

## Author Contributions

SB and AA conceptualized the study. SB, AA, JD, and SK implemented it. DRS supervised the whole project and contributed as PI. BR analyzed the data, designed maps and figures, drafted the initial version of manuscript, significantly contributed in editing, and revision of the manuscript. All authors contributed to the article and approved the submitted version.

## Funding

The study received support from the World Health Organization, Health Ethics & Governance Unit through the Public Health Emergency Preparedness and Response Ethics Network (PHEPREN). The WHO was not involved in the design or conduct of the study, nor in writing this report.

## Conflict of Interest

The authors declare that the research was conducted in the absence of any commercial or financial relationships that could be construed as a potential conflict of interest.

## Publisher's Note

All claims expressed in this article are solely those of the authors and do not necessarily represent those of their affiliated organizations, or those of the publisher, the editors and the reviewers. Any product that may be evaluated in this article, or claim that may be made by its manufacturer, is not guaranteed or endorsed by the publisher.

## References

[1] WHO. Coronavirus Disease (COVID-19) – Epidemiological Update. (2022). Available online at: https://www.who.int/emergencies/diseases/novel-coronavirus-2019/situation-reports (accessed December 2, 2021).

[2] Dahal S Luo R Subedi RK Dhimal M Chowell G. Transmission dynamics and short-term forecasts of COVID-19: Nepal 2020/2021. Epidemiologia. (2021) 2:639–59. 10.3390/epidemiologia2040043 PMC9620946 36417221

[3] Basnet BB Bishwakarma K Pant RR Dhakal S Pandey N Gautam D. Combating the COVID-19 Pandemic: Experiences of the First Wave From Nepal. Front Public Health. (2021) 9:613402. 10.3389/fpubh.2021.613402 34322466 PMC8310916

[4] Neupane P Bhandari D Tsubokura M Shimazu Y Zhao T Kono K. The Nepalese health care system and challenges during COVID-19. J Global Health. (2021) 11:1–3. 10.7189/jogh.11.03030 33692885 PMC7914404

[5] Rayamajhee B Pokhrel A Syangtan G Khadka S Lama B Rawal LB. How Well the Government of Nepal Is Responding to COVID-19? An experience from a resource-limited Country to confront unprecedented pandemic. Front Public Health. (2021) 9:85. 10.3389/fpubh.2021.597808 33681124 PMC7925847

[6] Ministry of Health and Population N. COVID-19 Dash Board. (2021). Available online at: https://covid19.mohp.gov.np/ (accessed January 10, 2022).

[7] Post TK. Nepal COVID-19 Updates. (2021). Available online at: https://kathmandupost.com/covid19 (accessed January 29, 2022).

[8] Sharma G. Nepal detects first two cases of Omicron variant - health ministry. Reuters. (2021). Available online at: https://www.reuters.com/world/asia-pacific/nepal-detects-first-two-cases-omicron-variant-health-ministry-2021-12-06 (accessed December 10, 2021).

[9] Weissenbach B. COVID-19 spirals out of control in Nepal: Every emergency room is full now. Natl Geogr Mag. (2021). Available online at: https://www.nationalgeographic.com/culture/article/a-pandemic-surge-threatens-livelihoods-in-nepal (accessed May 14, 2021).

[10] Shakya DR Thapa SB Kar SK Sharma V Uchida N Ortiz MR. COVID-19 across countries: situation and lessons for pandemic control. J BP Koirala Institute of Health Sciences. (2020) 3:9–27. 10.3126/jbpkihs.v3i1.30311

[11] GDP per capita (current US$) - Nepal. (2021). Available online at: https://data.worldbank.org/indicator/NY.GDP.PCAP.CD?locations=NP (accessed January 14, 2022).

[12] Bhattarai S Dhungana J Ensor T Shrestha UB. Assessment of service availability and Infection prevention measures in hospitals of Nepal during the transition phase of COVID-19 case surge. medRxiv. (2020). 10.1101/2020.05.13.20097675

[13] Shakya DR Shrestha RR Koirala S Kafle SU Subedi P Anand A. Social responsibility for health during COVID-19 pandemic. J BP Koirala Institute of Health Sciences. (2021) 4:48–55. 10.3126/jbpkihs.v4i1.3609133921512

[14] Lama P. Kathmandu's private hospitals fleece Covid-19 patients, multiplying crisis. Onlinekhabar. (2021). Available online at: https://english.onlinekhabar.com/kathmandus-private-hospitals-fleece-covid-19-patients-multiplying-crisis.html (accessed May 11, 2021).

[15] (NMC) NMC. Interim Clinical Guidance for care of patients with COVID-19 in healthcare settings. (2021). Available online at: https://nmc.org.np/interim-clinical-guidance-for-care-of-patients-with-covid-19-in-healthcare-settings-3 (accessed January 4, 2022).

[16] McGuire AL Aulisio MP Davis FD Erwin C Harter TD Jagsi R. Ethical challenges arising in the COVID-19 pandemic: an overview from the Association of Bioethics Program Directors (ABPD) task force. Am J Bioethics. (2020) 20:15–27. 10.1080/15265161.2020.1764138 32511078

[17] Kooli C. COVID-19: Public health issues and ethical dilemmas. Ethics Med Public Health. (2021) 17:100635. 10.1016/j.jemep.2021.100635 33553555 PMC7847409

[18] Behrens KG. Clinical Ethical Challenges in the COVID-19 Crisis in South Africa. Wits J Clin Med. (2020)2(SI):29–32. 10.18772/26180197.2020.v2nSIa534330365

[19] Peter Gill JRS. COVID-19: Nepal in Crisis. The Doiplomat. (2020). Available online at: https://thediplomat.com/2020/06/covid-19-nepal-in-crisis (accessed December 15, 2021).

[20] Subedi N. Ethical challenges in medical practice in the context of Coronavirus disease 2019 in Nepal. J of Gandaki Medical College - Nepal. (2020) 13:1–3. 10.3126/jgmcn.v13i1.29257

[21] Government of Nepal MoHaP. Nepal Health Sector Emergency Response Plan COVID-19 Pandemic. (2021). Available online at: https://mohp.gov.np/eng/about-us/divisions/health-emergency-disaster-management (accessed January 22, 2022).

[22] Kapiriri L. Medical ethics and bedside rationing in low-income countries: challenges and opportunities. Bioethics-medical, ethical and legal perspectives InTech: Croatia. (2016):199–213. 10.5772/65089

[23] Fletcher AJ Marchildon GP. Using the Delphi method for qualitative, participatory action research in health leadership. Int J Qualit Methods. (2014) 13:1–18. 10.1177/160940691401300101

[24] Farrell TW Francis L Brown T Ferrante LE Widera E Rhodes R. Rationing limited healthcare resources in the COVID-19 era and beyond: ethical considerations regarding older adults. J Am Geriatr Soc. (2020) 68:1143–9. 10.1111/jgs.16539 32374466 PMC7267288

[25] Dove ES Kelly SE Lucivero F Machirori M Dheensa S Prainsack B. Beyond individualism: Is there a place for relational autonomy in clinical practice and research? Clin Ethics. (2017) 12:150–65. 10.1177/1477750917704156 28989327 PMC5603969

[26] Mackenzie C Stoljar N. Relational Autonomy: Feminist Perspectives on Autonomy, Agency, and the Social Self. New York, NY: Oxford University Press (2000).

[27] Tanveer F Khalil AT Ali M Shinwari ZK. Ethics, pandemic and environment; looking at the future of low middle income countries. Int J Equity Health. (2020) 19:1–12. 10.1186/s12939-020-01296-z 33059674 PMC7557237

[28] Bansal P Bingemann TA Greenhawt M Mosnaim G Nanda A Oppenheimer J. Clinician wellness during the COVID-19 pandemic: extraordinary times and unusual challenges for the allergist/immunologist. J Allergy Clin Immunol. (2020) 8:1781–90. e3. 10.1016/j.jaip.2020.04.001 32259628 PMC7129776

[29] Chen Q Liang M Li Y Guo J Fei D Wang L. Mental health care for medical staff in China during the COVID-19 outbreak. Lancet Psychiatry. (2020) 7:e15–e6. 10.1016/S2215-0366(20)30078-X 32085839 PMC7129426

[30] Wicclair M. Allocating Ventilators During the COVID-19 Pandemic and Conscientious Objection. Amn j Bioethics. (2020) 20:204–7. 10.1080/15265161.2020.1777347 32716798

[31] Robert R Kentish-Barnes N Boyer A Laurent A Azoulay E Reignier J. Ethical dilemmas due to the COVID-19 pandemic. Ann Intensive Care. (2020) 10:1–9. 10.1186/s13613-020-00702-7 32556826 PMC7298921

[32] Asante AD Zwi AB. Factors influencing resource allocation decisions and equity in the health system of Ghana. Public Health. (2009) 123:371–7. 10.1016/j.puhe.2009.02.006 19364613

[33] WHO. Coronavirus disease (COVID-19): Ethics, resource allocation and priority setting. (2020). Available online at: https://www.who.int/emergencies/diseases/novel-coronavirus-2019/question-and-answers-hub/coronavirus-disease-covid-19-ethics-resource-allocation-and-priority-setting.

[34] Vindrola-Padros C Andrews L Dowrick A Djellouli N Fillmore H Bautista Gonzalez E. Perceptions and experiences of healthcare workers during the COVID-19 pandemic in the UK. BMJ Open. (2020) 10:e040503. 10.1136/bmjopen-2020-040503 33154060 PMC7646318

[35] Adhikari R. Ethics in undergraduate medical courses in Nepal. Kathmandu Univ Med J. (2013) 11:1–3. 10.3126/kumj.v11i1.11014 23774403

[36] Miyasaka M Akabayashi A Kai I Ohi G. An international survey of medical ethics curricula in Asia. J Med Ethics. (1999) 25:514–21. 10.1136/jme.25.6.514 10635508 PMC479305

[37] Coumare VN Pawar SJ Manoharan PS Pajanivel R Shanmugam L Kumar H. COVID-19 Pandemic-Frontline Experiences and Lessons Learned From a Tertiary Care Teaching Hospital at a Suburban Location of Southeastern India. Front Public Health. (2021) 9:673536. 10.3389/fpubh.2021.673536 34178928 PMC8232226

[38] Das SK Ranabhat K Bhattarai S Karki KB Gyanwali P Paneru HR. Combination of convalescent plasma therapy and repurposed drugs to treat severe COVID-19 patient with multimorbidity. Clin Case Reps. (2021) 9:2132–7. 10.1002/ccr3.3964 33821192 PMC8013972

[39] Wright KS. Ethical research in global health emergencies: making the case for a broader understanding of 'research ethics'. Int Health. (2020) 12:515–7. 10.1093/inthealth/ihaa053 33165558 PMC7651111

[40] Bioethics NCo. Research in Global Health Emergencies: Ethical Issues. Nuffield Council on Bioethics (2020). Available online at: https://www.nuffieldbioethics.org/assets/pdfs/RGHE_Short_report_web_version1.pdf (accessed January 25, 2022).

[41] Singh DR Sunuwar DR Shah SK Karki K Sah LK Adhikari B. Impact of COVID-19 on health services utilization in Province-2 of Nepal: a qualitative study among community members and stakeholders. BMC Health Serv Res. (2021) 21:1–14. 10.1186/s12913-021-06176-y 33627115 PMC7903406

[42] Grabowski DC Mor V. Nursing home care in crisis in the wake of COVID-19. J Am Med Assoc. (2020) 324:23–4. 10.1001/jama.2020.8524 32442303

[43] Solbakk JH Bentzen HB Holm S Heggestad AKT Hofmann B Robertsen A. Back to WHAT? The role of research ethics in pandemic times. Med Health Care Philoss. (2021) 24:3–20. 10.1007/s11019-020-09984-x 33141289 PMC7607543

[44] COVID treatment in private hospitals not accessible to all Nepal. (2021). Available online at: https://thehimalayantimes.com/nepal/covid-treatment-in-private-hospitals-not-accessible-to-all.

[45] Rubinelli S Myers K Rosenbaum M Davis D. Implications of the current COVID-19 pandemic for communication in healthcare. Patient Educ Couns. (2020) 103:1067. 10.1016/j.pec.2020.04.021 32451002 PMC7243771

[46] Lippi D Bianucci R Donell S. Role of doctors in epidemics: historical perspectives and implications for COVID-19. Int Emerg Med. (2020) 15:883–4. 10.1007/s11739-020-02351-x 32390080 PMC7211268

[47] Baines P Draper H Chiumento A Fovargue S Frith L. COVID-19 and beyond: the ethical challenges of resetting health services during and after public health emergencies. J Med Ethics. (2020) 46:715–6. 10.1136/medethics-2020-106965 33067314 PMC7656144

