# Supplementary material for: Participatory Approach to Develop Evidence-Based Clinical Ethics Guidelines for the Care of COVID-19 Patients: A Mixed Method Study From Nepal
Source: Front Public Health. 2022 Jun 27;10:873881. doi: 10.3389/fpubh.2022.873881 (PMC9272001; doi:10.3389/fpubh.2022.873881)
Supplement: Supplementary file 1 [file Data_Sheet_1.pdf]

Supplementary file 1

1.1 STUDY QUESTIONNAIRE (ACTIVITY I)

Part I: Background

1. Gender:

a) Male ☐
b) Female ☐
c) Other ☐

2. Age: _________Years

3. Where did you obtain your medical training from?

a) Undergraduate: Country __________ Medical school ___________________
b) Post graduate: Country ___________ Medical school ___________________

4. Since graduation, how many years have you been working in medical sector? _____ years

5. Are you currently working as a

a) General Practice/ Family medicine physician
b) Resident in (please specify) _________
c) Specialist in (please specify) _________
d) Nurse (please specify department) ___________
e) Health Assistant (please specify department) ___________
f) Public health officer (please specify department) ___________
g) Others (please specify department) ___________

6. What is the type of your primary work institution?

(you can check more than one if applicable, *put X mark to indicate your selection*)?

a) Government institution
b) Private nonprofit institution
c) Private for profit institution
d) Own private institution
e) Private wing in a government facility
f) Others___________

7. On an average how many hours per week do you spend in

a) Government institution ____
b) Private wing______
c) Private institution______

8. On an average how many patients do you provide service to in a week (both inpatient and outpatient)? _____________

Part II: Ethical Dilemmas

1. **Please *put X mark* to indicate your selected score under each subheading challenges (that you might have confronted during COVID-19 pandemic March 2020- Jan 2021) based on the frequency of being 'never encountered (0)' to 'always encountered (5)'.**

Types of challenges	Score					
**a. Contextual challenges**	0	1	2	3	4	5
Resource scarcity (health systems related)						
Patients' socioeconomic status (poverty)						
**b. Challenges in decision making process**						
Perceived lack of training in resource allocation ethics						
Perceived lack/inadequacy of guidelines						
Disagreements among professionals						
Choice of patients to restrict access to treatment						
Balancing patient and family interests						
Lack of credible evidence						
No forum for discussion ethical issues						
**c. Provider-related challenges**						
Perceived lack of control of the decision yet held responsible						
Feelings of helplessness and incompetence						
Use of unacceptable criteria and how to deal with it						
Dual role of the physicians						
Witnessing and feeling responsible for the consequences of rationing for the patient						
Moral distress						
**d. Patient-related challenges**						
Inability to communicate due to lack of knowledge						
Differing decisions to doctors						
Excessive demand by "able" patients						

0= Never

1= Seldom

2= Sometimes

3= Moderately relevant

4= Frequently

5= Always

Adapted from doi: 10.5772/65089

2. **This session includes five open-ended questions that all participants are supposed to describe their opinions on how they would have handled these challenges OR even if they experienced these challenges how did they deal with?**

a. You were restricting treatment to a non-COVID-19 patient to give those resources to someone with COVID-19 who could benefit more (i.e. hospital bed, ventilator, medication). However, there was significant disagreement among health care personnel on commencing treatment of the COVID-19 patient due to lack of resources, what would you do and why? What ethical principles would you perceive to be important in this context?

b. The preferred course of COVID-19 treatment is not pursued because the patient is neither able to pay, nor is enrolled with any health insurance schemes or programs (public or private), what would you do and why? What ethical principles would you perceive to be important in this context?

c. Your preferred course of treatment conflicted with institutional policies, professional codes of ethics or laws. What would you do and why? What ethical principles would you perceive to be important in this context?

d. You came across colleagues not providing appropriate COVID-19 care because of their inadequate medical knowledge and skills, what would you do and why? What ethical principles would you perceive to be important in this context?

e. You witnessed that a colleague was not acting according to the professional standards during COVID-19 care (like not being honest, fair, responsible and respectful), what would be your reaction and why? What ethical principles would you perceive to be important in this context?

f. Any other issue that you would like to bring up and discuss?

1.2 PARTICIPANT FEEDBACK FORM (ACTIVITY IV)

Title of training	Location	Trainer	Date

Provide a Rating for each of the statements, below, by placing an "X" in the corresponding box.	**STRONGLY DISAGREE**	**DISAGREE**	**NEUTRAL**	**AGREE**	**STRONGLY AGREE**
The objectives of the training were defined well beforehand.					
Participation was encouraged throughout the training.					
The topics covered were relevant and informational.					
The materials and content were well chosen and helpful.					
The training will be helpful to my own work.					
The trainer was knowledgeable about the subject matter.					
The trainer was well prepared and thorough.					
The time allotted for the training was sufficient.					
The training location was well chosen.					

Additional comments
